# Grand challenges in genetics and epidemiology of allergic diseases: from genome to exposome and back

**DOI:** 10.3389/falgy.2024.1368259

**Published:** 2024-02-05

**Authors:** Luis Garcia-Marcos

**Affiliations:** Paediatric Allergy and Pulmonology Units, IMIB Bio-Medical Research Institute, Virgen de la Arrixaca University Children’s Hospital, University of Murcia, Murcia, Spain

**Keywords:** genetics, allergy, epidemiology, asthma, rhinoconjunctivitis, eczema

## 1 Epidemiology

### 1.1 Surveillance

Although many epidemiological studies searching for clues with the aim of disentangling the origins of allergic diseases have been conducted in many parts of the world for decades, the task of surveillance at a global level—fundamental for establishing such basic facts as whether prevalence is changing—has mainly been carried out by the International Study of Asthma and Allergies in Childhood (ISAAC) since 1998 (1) and subsequently by the Global Asthma Network (GAN) (2). Although the reports arising from that collaboration indicate a stable trend in the prevalence of asthma and rhinitis over the last decade (3, 4) and a certain increase in atopic eczema (5), there is a clear need for continuing surveillance at a global level using the same methods and involving as many centres as possible worldwide in order to establish whether those trends are ongoing or changing.

Despite the use of very basic investigative tools, such findings can establish important key details in the search for the origin, and thus approaches to prevention, of allergic diseases. A very good example is the dramatic increase in the prevalence of these diseases that occurred in the second half of the past century. This rapid increase indicates that the changes cannot have a purely genetic origin and that the interaction between the modifying environment and the individual genome, which may make some people more prone to disease, should be considered (6, 7). This consideration is mainly related to the more frequent atopic diseases and may not be so applicable to others, the origins of which might be different. This could be the case in the case of chronic spontaneous urticaria, which seems to be more related to autoimmune disease than to atopy (8). In this context, the question of why the prevalence of anaphylaxis seems to be on the rise is puzzling (9) and deserves future investigation.

### 1.2 Affordability

Surveillance at a global level is also fundamental in establishing how allergic diseases are managed and identifying the needs of countries with lower income in enabling access to the right medicines. Although we have guidelines for treatment everywhere, it is recognized that they are not followed in many instances (10) for several reasons, including the lack of affordability in low-resource settings (11). Is there a way that patients with severe disease can obtain access to biological treatments in low-income countries? Unfortunately, this is a very remote vision due to their cost in the market (12). Still, epidemiology remains crucial in pushing health authorities to implement policies to address this issue: unless the size of the problem is known and politicians are forced to face up to it, the right policies will never be carried out.

### 1.3 Risk factors and causality

The number of epidemiological studies dealing with risk factors for allergic diseases is enormous, and the literature attempting to summarise the results is also immense. However, a very small number of factors have been indisputably established to have causal associations with allergic diseases. For instance, only smoking in pregnancy seems to be a “universal” factor leading to asthma in childhood (13) even across generations (14) and could be considered a cause (13, 15). Quite probably, and as has been claimed, what provokes the inception of allergic diseases is probably not individual causes acting on individuals; rather, a conglomeration of factors interacting with one another and with the individual is what ends up causing allergic diseases, directly or through epigenetic changes. The package classically called “westernization”, which includes factors such as pollution, food, stress, and hygiene, among others, is a perfect example (16).

### 1.4 Prediction and prevention

Knowledge of causal factors that predict allergic diseases can put us in a position to design studies to identify methods of preventing those diseases. Perhaps the best example is the one derived from the hygiene hypothesis, recently extended to dysbiosis modulation (17) or Bayesian interpretations (18). In the case (again) of asthma, the administration of bacterial lysates seems (19, 20) to be a field in which clinical trials are needed to clarify whether an inexpensive intervention can make a major difference. These potential trials are probably not on the radar of the pharmaceutical industry, as bacterial lysates are probably not good business.

However, it is not a matter of just primary prevention but also one of secondary prevention, i.e., preventing flare-ups of disease, especially of a more severe form. Preventing severe cases implies, again, predicting those cases. The study of phenotypes and endotypes of allergic diseases and how to predict them (21) can help in choosing the most efficacious approach to prevention and management. Genetics is fundamental in this respect (22).

## 2 Genetics

### 2.1 A failure?

Although enormous efforts and funds have been invested in disentangling the genetic basis of asthma and allergic diseases, and although great advances in the basic research field have been achieved, there had not been much change in the clinical domain over the past two decades until the market inception of new biologic drugs. Some of these drugs, of course, could have been developed after the discovery of immunological pathways, which have been pointed out after the discovery of their association with certain genetic polymorphisms with predisposition to specific allergic diseases or atopy (23). Although much more was expected from the study of the genetics of allergic diseases, these efforts cannot be regarded as having been wasted. As mentioned, the discovery of polymorphisms associated with these diseases has shed some light on their molecular mechanisms, helping to define phenotypes and endotypes.

### 2.2 Expansion to different ancestries

Most genetic studies carried out so far have been performed in populations of European ancestry, and studies of African-American and Latino/Hispanic populations are still underpowered. The higher genetic heterogeneity in these populations means that these studies will probably demand larger sample populations than required for those carried out in European populations. It is quite probable that findings in GWASs in European populations will be replicated in non-European ones, but more and larger studies are needed (24). GWASs in different ethnicities can also help to better define phenotypes and design tailored treatments.

### 2.3 Epigenetics

Following the linkage analyses and the GWAS era in which associations were established, many of these based on a limited understanding of the processes involved, we are probably now in the midst of a period of study of “regulation”. We are trying to understand how certain genes are switched on or off, either by other genes or through interaction with the environment via epigenetic mechanisms (25). We are also trying to comprehend how the transcription of such genes is made and potentially modified (21).

It is in this domain that epigenetics has taken centre stage in explaining why the incidence of allergic diseases has increased so much over a relatively short period of time, as this does not seem to have a genetic explanation. It seems, therefore, that the interaction between genes and the environment will be the key research topic in years to come. As Bellanti has put it, “genetics loads the gun and epigenetics pulls the trigger” (26). For the convenience of this context in which epidemiology and genetics are linked, it might be put in a slightly different way: genetics is the bullet, epigenetics the trigger and firing mechanism, and the environment the hand that pulls the trigger. The conditions must be suitable for all three drivers for shooting to occur.

The field open in front of us is now broader, and so far, the information obtained is inadequate for us to draw strong conclusions. For instance, in a recent systematic review of the genetic, epigenetic, and environmental factors related to allergic rhinitis, Albloushi and Al-Ahmad (27) conclude that more studies examining specific factors relating to the aforementioned drivers are needed to make it possible to compare results between studies and to start drawing conclusions that can lead to the investigation of specific drugs that can provide more closely tailored treatment.

Matters can become even more complicated when certain polymorphisms are related to allergic diseases in some ethnicities and not in others (maybe due to environmental factors related to those ethnicities). For instance, Dastgheib et al. (28) performed a stratified meta-analysis on the association between certain polymorphisms of the IL-10 gene and pediatric asthma. Although their main conclusion was that none of these (IL-10-1082G > A, −819C > T, or −592C > A) were associated with asthma in the overall population, there was a significant association of IL-10-1082G > A with pediatric asthma in Asian and Chinese populations.

## 3 Basic common requirements of epidemiology and genetics

### 3.1 Definition of case

Both in epidemiology and in genetics, the way in which the endpoint is defined is fundamental. While some allergic diseases are relatively easily defined and their severity graded (for instance, atopic dermatitis), others, such as asthma, shown considerable variation in phenotypes, especially in the pediatric ages. In addition to making the results of different studies more difficult to compare, this variability adds noise to the processing of potential large collaborative datasets, and creates even greater difficulty for attempts at meta-analysis of currently available data. This was the case for the attempt by Fan et al. to summarise results on the association between nitric oxide synthase gene variants and pediatric asthma (29). They found that although some associations between these variants and the prevalence and outcomes of asthma were present, these associations varied according to the type of variant, race, study design employed, and disease definition.

Even in the cases of some allergic diseases that can (apparently) be defined more clearly as compared to asthma, specific objective measurements that can be used to better characterize the disease, such as trans-epidermal water loss (TEWL) in atopic dermatitis, may be not entirely reliable. Green et al. (30) studied various factors that could affect TEWL values and concluded that at least 12 were not well controlled in experimental settings, which led them to conclude that the definition of normal TEWL is probably problematic. However, even when external factors can be fully controlled, as may be the case with TEWL, genetic factors can alter the response to drugs used in the process of definition of the disease. This is the case in the bronchial dilation test in asthma: certain polymorphisms of the adrenoceptor β2 (ADRB2) gene are better able to respond to inhaled β2 agonists (31).

The “Faustian bargain” needs to be considered here, as it is not always the case that “bigger is better” (32); larger studies can introduce more noise and confusion, hiding findings that might have been revealed if the sample was more smaller but more homogeneous.

### 3.2 Data analyses: systems biology and big data

The plethora of information obtained from the epidemiological and genetic fields is still growing and will continue to grow in the future. The need for new ways to process this information puts artificial intelligence and systems biology in the nucleus of the new era (33). There is a need for a close collaboration between allergists, basic scientists, and professionals working in artificial intelligence to create algorithms that help to provide an understanding of the data and make them useful for prevention, diagnosis, and treatment. Avoiding confusion and overcoming the limitations of artificial intelligence (bias, harm, ethical considerations, regulations, etc.) is a major challenge that we are beginning to face (34).

### 3.3 Alternative hypotheses

Systems biology and artificial intelligence can discover niches that might be unknown or forgotten. Although type 2 inflammation has been regarded as the backbone of allergy and certain asthma phenotypes for many years, we must be open to other mechanisms that might be complementary to the best known currently. An example is the epithelial barrier hypothesis, which proposes that the dramatic increase in allergic diseases in recent decades might be explained by the increase in barrier-damaging agents as a result of “westernization” (35, 36). In asthma, another example is the forgotten notion that smooth muscle “disuse contracture” might be part of the condition, at least in some phenotypes (37). Open-mindedness and intellectual humility are crucial in achieving advancement, especially when research is multidisciplinary (38).

## Author contributions

LG-M: Writing – original draft, Writing – review & editing.
